# A chemically-defined growth medium to support *Lactobacillus-Acetobacter sp.* community analysis

**DOI:** 10.1371/journal.pone.0292585

**Published:** 2023-10-12

**Authors:** Kevin Aumiller, Robert Scheffler, Eric T. Stevens, Zehra T. Güvener, Emily Tung, Anna B. Grimaldo, Hans K. Carlson, Adam M. Deutschbauer, Michiko E. Taga, Maria L. Marco, William B. Ludington

**Affiliations:** 1 Department of Embryology, Carnegie Institution of Washington, Baltimore, MD, United States of America; 2 Department of Biology, Johns Hopkins University, Baltimore, MD, United States of America; 3 Department of Food Science and Technology, University of California, Davis, Davis, CA, United States of America; 4 Molecular and Cell Biology Department, University of California, Berkeley, Berkeley, CA, United States of America; 5 Department of Plant and Microbial Biology, University of California, Berkeley, Berkeley, CA, United States of America; 6 Lawrence Berkeley National Laboratory, Department of Environmental Genomics and Systems Biology, Berkeley, CA, United States of America; Fonterra Coop / Lebanese University, LEBANON

## Abstract

Lactobacilli and *Acetobacter sp.* are commercially important bacteria that often form communities in natural fermentations, including food preparations, spoilage, and in the digestive tract of the fruit fly Drosophila melanogaster. Communities of these bacteria are widespread and prolific, despite numerous strain-specific auxotrophies, suggesting they have evolved nutrient interdependencies that regulate their growth. The use of a chemically-defined medium (CDM) supporting the growth of both groups of bacteria would facilitate the identification of the molecular mechanisms for the metabolic interactions between them. While numerous CDMs have been developed that support specific strains of lactobacilli or *Acetobacter*, there has not been a medium formulated to support both genera. We developed such a medium, based on a previous CDM designed for growth of lactobacilli, by modifying the nutrient abundances to improve growth yield. We further simplified the medium by substituting casamino acids in place of individual amino acids and the standard Wolfe’s vitamins and mineral stocks in place of individual vitamins and minerals, resulting in a reduction from 40 to 8 stock solutions. These stock solutions can be used to prepare several CDM formulations that support robust growth of numerous lactobacilli and *Acetobacter*s. Here, we provide the composition and several examples of its use, which is important for tractability in dissecting the genetic and metabolic basis of natural bacterial species interactions.

## Introduction

Lactic Acid Bacteria (LAB) and Acetic Acid Bacteria (AAB) coexist in nature in a wide variety of environments [1]. These include food fermentations such as wine [1], beer [2], kefir [3, 4], sauerkraut [5], kimchi [6], bread [7], and cacao beans [8]. They also have been found together in agricultural feed, such as silage [9]. LAB and AAB can coexist as well in mammalian and insect gastrointestinal tracts where they have probiotic benefits [10]. This includes the intestine of the genetic model animal *Drosophila melanogaster*, where lactobacilli and *Acetobacter sp.* are the core types of LAB and AAB, respectively [11]. We note that due to the recent reclassifications within the former *Lactobacillus* genus [12], we refer to the LABs used in this study by their newly given formal scientific names, *Lactiplanibacillus plantarum* (*Lp. plantarum*) and *Levilactobacillus brevis* (*Ll. brevis*) or collectively by their common name, lactobacillus (plural lactobacilli).

Why LAB and AAB co-occur so prevalently is the subject of ongoing investigations. A synergistic metabolism between lactobacilli and *Acetobacter*s has been demonstrated based on sharing of organic short chain fatty acids (SCFAs), including lactic acid and acetic acid. These compounds are generally known to mutually promote growth through cross-feeding, such that lactate stimulates *Acetobacter* growth and acetate stimulates lactobacilli [13, 14]. There is biomedical relevance of SCFA as their production by the gut microbiome plays a key role in gut epithelial cell metabolism and immune homeostasis [15, 16] potentially underlying the role of LABs as probiotics [10]. Furthermore, in the mammalian colonic crypts, microbial assemblages include lactobacilli and other Lactobacillales such as *Streptococcus*, and *α*-Proteobacteria besides *Acetobacter*, including *Sphingomonas* and *Paracoccus* [17, 18], suggesting there may be a broader phylogenetic pattern of co-existence for these groups. Studying these relationships in synthetic microbial communities could provide insights into their roles in more complex environments with multiple trophic levels such as the digestive tract. For instance, cross-feeding may extend beyond SFCAs to other metabolites, namely B-vitamins, which are known to impact host health [19, 20]. Cross-fed nutrients could also influence secondary metabolite production [21]. Thus, there exists a pressing need to study the molecular mechanisms of metabolic interactions in natural gut microbial communities, and the lactobacilli-*Acetobacter* communities in the *Drosophila* gut are a promising model.

Identifying the nutrient requirements for organismal growth [22] as well as the metabolites produced [23] during fermentation is greatly improved with the use of chemically-defined growth media (CDM). Various CDM recipes have been developed to support growth of lactobacilli [24–30], yet no efforts have been made to develop a CDM with the express purpose of supporting lactobacillus-*Acetobacter* communities for microbiology experiments. As a resource to investigate the mechanisms of lactobacillus-*Acetobacter* nutritional cross-feeding, we developed a chemically-defined medium (CDM) that independently supports growth of several *Lp. plantarum* strains. By altering the nutrient composition, we show that the CDM can enable growth of both lactobacilli and *Acetobacter sp.* isolated from the fruit fly gut microbiome and other sources in high throughput. Therefore, this CDM fulfills the purpose of growing lactobacilli and *Acetobacter sp.* under chemically-defined conditions, which will enable experiments to determine the molecular and genetic mechanisms of their nutrient exchange.

We based this medium on the formulation of [29]. Our medium may be modified to optimize the growth of either lactobacilli or *Acetobacter*s to a density of ∼ 10^9^ cells/mL (OD_600_ > 1) or to support co-cultures. In this short report, we provide the chemical composition of the medium, some guides on its preparation, an approach to circumvent strain-specific auxotrophies, and some known issues that can result from chemical impurities. We focus our results on the lactobacilli and *Acetobacter sp.* from the *Drosophila* gut microbiome.

## Materials and methods

### Chemicals

A complete list of all chemicals used in the various CDM formulations is provided in S1 Table in S1 File.

### Media preparation

Stock solutions were individually prepared in ultra-pure water or in appropriate solvents as indicated in Table 1. All stock solutions were passed through 0.22 *μ*m filter and kept in dark at 4°C except nucleotides, which were stored at -20°C. FeSO_4_ ⋅ 7H_2_O was freshly prepared. Medium was initially made with 30% less water to allow customized additions. Final pH of CDM was adjusted to 6.5 and the medium was passed through a 0.22 *μ*m filter. CDM was stored at 4°C and used within 2 days.

**Table 1:** Composition of the chemically-defined growth medium.

Compound	Concentration	Units	Supplier	Part Number	Stock
**Base components**					
MOPS	40	mM	Millipore	475898	10x in H_2_O
K_2_HPO_4_	5	mM	Fisher	P288	10x in H_2_O
NaCl	0.2	mM	Fisher	S271	100x in H_2_O
NH_4_Cl	20	mM	Fisher	A649	100x in H_2_O
K_2_SO_4_	10	mM	Sigma	746363	50x in H_2_O
MgCl_2_.6H_2_O	1	mM	Fisher	BP214	100x in H_2_O
MnCl_2_.4H_2_O	0.05	mM	Sigma	M3634	100x in H_2_O
FeSO_4_.7H_2_O	0.05	mM	Sigma	F8633	100x in H_2_O
**Amino acids**					
L-Alanine	14	mM	Sigma	A7627	40x in H_2_O
L-Arginine	0.36	mM	Sigma	A5131	200x in H_2_O
Glycine	3.41	mM	Sigma	G7126	200x in H_2_O
L-Lysine	3.59	mM	Sigma	L5626	200x in H_2_O
L-Proline	1.737	mM	Sigma	P0380	200x in H_2_O
L-Histidine	2.664	mM	Sigma	H8125	200x in H_2_O
L-Serine	7.374	mM	Sigma	S4500	200x in H_2_O
L-Threonine	2.098	mM	Sigma	T8625	200x in H_2_O
L-Aspartic acid	0.083	mM	Sigma	A9256	200x in 1 M HCl
L-Asparagine	4.162	mM	Sigma	A0884	200x in 1 M HCl
L-Tyrosine	1.1035	mM	Sigma	T3754	200x in 1 M NaOH
L-Cysteine-HCl	4.758	mM	Sigma	C1276	200x in H_2_O
L-Valine	4.268	mM	Sigma	V0500	200x in 1 M NaOH
L-Glutamic acid	1.417	mM	Sigma	G1251	200x in 1 M HCl
L-Tryptophane	1.371	mM	Sigma	T0254	200x in 1 M NaOH
L-Phenylalanine	1.513	mM	Sigma	P2126	200x in 1 M HCl
L-Glutamine	3.267	mM	Sigma	G3126	200x in 1 M NaOH
L-Leucine	1.905	mM	Sigma	L8000	2000x in 0.5 M HCl
L-Isoleucine	1.905	mM	Sigma	I2752	2000x in 0.5 M HCl
L-Methionine	0.67	mM	Sigma	M9625	200x in 0.1 M HCl
**Nucleotides**					
Guanine	0.033	mM	Sigma	G6779	200x in 0.1 M NaOH
Uracil	0.0445	mM	Sigma	U1128	200x in 0.1 M NaOH
Xanthine	0.0325	mM	Sigma	X4002	200x in 0.1 M NaOH
Adenine	0.037	mM	Sigma	A2786	200x in 0.1 M HCl
**Carbon**					
Glucose	125	mM	Sigma	G8270	50x in H_2_O
Acetate	10	mM	Fisher	S210	100x in H_2_O
D,L-Lactate	0.6	mM	Sigma	69785	100x in H_2_O
**Optional**					
Ascorbic acid	1.4	mM	Fisher	AA3623714	100x in H_2_O
Lipoic acid	1	mM	Sigma	T1395	direct
Fructose	62.5 or 125	mM	Sigma	F3510	50x in H_2_O

To make a carbon-free CDM, the following components were combined in the following order in a final volume of 17.5 mL: 8.375 mL ultra-pure water, 2.5 mL MOPS buffer, 0.25 mL K_2_HPO_4_, 0.25 mL NaCl, 0.25 mL NH_4_Cl, 0.5 mL K_2_SO_4_, 0.625 mL L-Alanine, 0.125 mL L-Arginine, 0.125 mL Glycine, 0.125 mL L-Lysine, 0.5 mL L-Proline, 0.125 mL L-Histidine, 0.125 mL L-Serine, 0.125 mL L-Threonine, 0.125 mL L-Aspartic acid, 20 uL of 10 N NaOH for pH correction, 0.125 mL L-Asparagine, 10 uL of 10 N NaOH for pH correction, 0.125 mL L-Tyrosine, 0.125 mL L-Cysteine-HCl, 0.125 mL L-Valine, 0.125 mL L-Glutamic acid, 0.125 mL L-Tryptophane, 0.125 mL L-Phenylalanine, 0.125 mL L-Glutamine, 0.125 mL L-Leucine, 0.125 mL L-Isoleucine, 0.125 mL L-Methionine, 0.125 mL Ca-D-(+)-pantothenate, 0.125 mL Lipoic acid, 0.125 mL Nicotinic acid, 0.125 mL para-Aminobenzoic acid, 0.125 mL Pyridoxine-HCl, 0.125 mL Thiamine-HCl, 0.125 mL Biotin, 0.125 mL Ascorbic acid, 0.125 mL Folic acid, 0.125 mL Guanine, 0.125 mL Uracil, 0.125 mL Xanthine, 0.125 mL Adenine, 0.25 mL MgCl_2_ ⋅ 6H_2_O, 0.25 mL MnCl_2_ ⋅ 4H2O, 0.25 mL FeSO_4_ ⋅ 7H2O.

Appropriate carbon sources were provided in CDM to grow specific isolates. A final concentration of 1% glucose and 0.1% acetate were added for the growth of *Lp. plantarum*. A final concentration of 1% glucose, 0.5% fructose and 0.1% acetate supported limited growth of *Ll. brevis*. A final concentration of 1% fructose was used for the growth of *A. pasteurianus*, and 1% glucose was used for the growth of *A. tropicalis*.

### Bacterial strains and growth

Bacterial strains used in this study are listed in S2 Table in S1 File. Prior to CDM experiments, strains were initially cultured in rich media to maximize growth rate. These include MRS medium for lactobacilli and Mannitol-Yeast Extract-Peptone medium supplemented with 1% DL-Lactic acid (MYPL) for *Acetobacter*s. Rich medium streak plates were routinely prepared from glycerol stocks and incubated at 30°C for 48 hours and used only once. From these plates, 2–3 mL rich media were inoculated with single colonies and grown at 30°C for 16 to 18 h. To facilitate efficient growth in CDM for growth kinetics experiments, cultures were passaged once in a 1:1 mixture of rich medium and CDM followed by 1–3 passages in CDM. Turbidity was then adjusted to OD_600_ = 1 and cultures were diluted 1:100 into 200 *μ*L CDM in optically clear 96-well microplates. Microplates were incubated at 30°C in a temperature-controlled plate reader either statically or with double orbital shaking at a speed of 425 CPM and an orbital radius of 3 mm. For static cultures, plates were subjected to linear shaking at 425 CPM for 10 seconds prior to each measurement to resuspend bacteria. OD_600_ measurements were taken every 60 minutes in static cultures and every 5–15 minutes in shaken cultures for 48 hours. Static culture plates were sealed with a low-evaporation plastic lid. Shaken culture plates were sealed with a Breath-Easy film, and off-center holes were poked over each well out of the way of the detector with a 16 gauge needle to ensure optimal aeration for *Acetobacter sp*. M1000 (Tecan), Magellan, and Epoch 2 (Biotek) plate readers were used. OD_600_ measurements were background-subtracted using the OD_600_ measurement of medium-only controls. Each experiment was performed for 12 experimental replicates from three biological replicates.

### Statistical analysis

Growth curves were smoothed with a cubic spline and standard error of the mean was calculated on a five time point window rolling average across the 12 replicates. All analysis was performed in MATLAB R2021b.

## Results and discussion

### Formulation of the CDM for lactobacillus growth

**Fig 1:**
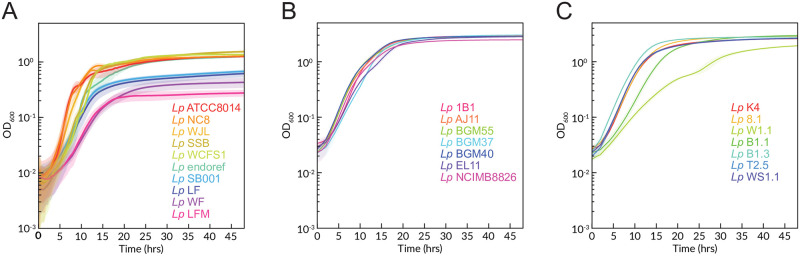
Growth of *Lactiplantibacillus plantarum* isolates in CDM_*L*_. Growth of *Lactiplantibacillus plantarum* isolates from different sources grown with variations of CDM_*L*_ in different laboratories (S2 Table in S1 File). **(A)** 10 isolates predominantly from *Drosophila* guts grown aerobically with continuous shaking in the CDM_*L*_ with a starting inoculum of 0.01 OD_600_. Note *Lp* WCFS1 is a human mouth isolate. **(B)** Seven isolates of *L. plantarum* from food fermentations and mammals grown anaerobically in CDM_*L*_, shaken intermittently before each OD reading. Note *Lp* WCFS1 in panel A is a single colony derivative of *Lp* NCIMB8826 in panel B. Both are from the same human mouth isolate. **(C)** Seven isolates of *L. plantarum* from food fermenatations grown anaerobically in a rich formulation of CDM_*L*_ with 10x L-cysteine. Solid lines represent means of 12 technical replicates. In panel A, Time point OD_600_ readings were taken every 5 minutes, and shaded areas represent standard error on a 5-point rolling average. for panels B and C, time point OD_600_ readings were taken every every 1 hour for panels B and C, and shaded areas represent standard error at each time point (note the error in B and C is very small except at early time points).

With the goal of formulating a CDM that supports growth of the lactobacilli and *Acetobacter* isolated from the fruit fly gut (see S2 Table in S1 File), we first tested a previous chemically-defined medium that was developed for lactobacilli [29]. This medium contains glucose, all amino acids, and nucleotides. It supported limited growth of lactobacilli to an OD_600_ of only approximately 0.2, and it precipitated upon storage. We eliminated precipitation by decreasing the amino acid concentrations to the levels described by Palmer *et el.* 2007 [31] and added 40 mM MOPS buffer (3-morpholinopropane-1-sulfonic acid). This modified medium supported growth of *Lp. plantarum* LFM, a *D. melanogaster* isolate, to an OD_600_ of ≈ 0.2 (S3 Table in S1 File), corresponding to ∼ 10^8^ cells/mL. This low value is ∼ 10-fold lower density than reported for the *Lp. plantarum* strain analyzed in the previous study [29]. As strain-specific differences in media preferences are widely reported in lactobacilli [32], we further refined the composition by individually adding amino acids, vitamins and nucleotides at 10-fold higher concentrations into the original CDM (S4 Table in S1 File). Higher amounts of tyrosine and cysteine increased media precipitation, causing a higher OD_600_ reading, which would interfere with high throughput growth measurements. Improved growth yield with excess tryptophan was reproducible, thus we increased the concentrations in the CDM to 1.4 mM, (Table 1). We also experimented with increasing or decreasing the total amino acids, vitamins, and nucleic acids (S3 Table in S1 File), which showed we could double these components to increase *Lp. plantarum* growth without substantially affecting other lactobacilli and *Acetobacter* species. We next simplified the medium to reduce the total number of stock solutions. We first replaced the vitamins and minerals with Wolfe’s vitamins and Wolfe’s minerals stocks. We then replaced the individual amino acids with casamino acids, which are a mixture of amino acids derived from acid hydrolysis of casein, having a representative abundance of every individual amino acid except tryptophan. This variant of the medium, named CDM_*L*_ still required additional alanine (14 mM) and cysteine (12 mM) (S1 Fig in S1 File; Table 2) but supported rapid growth of a variety of *Lp. plantarum* strains from different sources (Fig 1, S2 Table in S1 File) to an OD_600_ > 1, corresponding to ∼ 10^9^ cells/mL under aerobic (Fig 1A) and anaerobic conditions (Fig 1B and 1C).

**Table 2:** Composition of CDM_*L*_.

Stock solution #	Chemical	Final concentration (1x)
**Buffer and salts**		
1	MOPS	40 mM
1	K_2_HPO_4_	5 mM
1	NH_4_Cl	20 mM
1	Na_2_SO_4_	10 mM
**Metals**		
2	MgCl_2_ ⋅ 6 H_2_O	1 mM
2	MnCl_2_ ⋅ 4 H_2_O	0.05 mM
2	FeSO_4_ ⋅ 7 H_2_O	0.05 mM
**Carbon source**		
3	Glucose	125 mM
3	Ammonnium acetate	10 mM
**Amino acids**		
4	Casamino acids	3 g/L
5	Cysteine-HCl ⋅ H_2_O	0.145 g/L
6	Tryptophan	0.05 g/L
7	**Wolfe’s vitamins**	1x
8	**Wolfe’s minerals**	1x

**10x Wolfe’s vitamins** contains 10 mg/L Ca-(D)-(+)-pantothenate (Sigma C8731), 10 mg/L Nicotinic acid (Sigma N4126), 10 mg/L para-Aminobenzoic acid (Nutr. Biochem. R-238), 20 mg/L Pyridoxine-HCl (Alfa aesar A12041), 10 mg/L Thiamine HCl (EMD Millipore 5871), 4 mg/L Biotin (Sigma B4639) in 0.1 M NaOH, 4 mg/L Folic acid (Sigma F8758) in 0.1 M NaOH, 0.2 mg/L Vitamin B_12_ (TCI C0449). **10x Wolfe’s minerals** contains 3 g/L Nitrilotriacetic Acid (Sigma N9877), 6 g/L MgSO_4_ ⋅ 7 H_2_O (EMD Millipore MX0070–1), 1 g/L MnSO_4_ ⋅ H_2_O (Fisher M113), 2 g/L NaCl (Fisher S271), 0.2 g/L CaCl_2_ (Mallinckrodt 4160), 0.2 g/L FeSO_4_ ⋅ 7 H_2_O (Sigma F8633), 0.2 g/L CoCl_2_ ⋅ 6 H_2_O (Sigma 202185), 0.2 g/L ZnSO_4_ ⋅ 7 H_2_O (Fisher Z68), 20 mg/L CuSO_4_ ⋅ 5 H_2_O (VWR 330), 20 mg/L AlK(SO)_4_ ⋅ 12 H_2_O (Sigma 237086), 20 mg/L H_3_BO_3_ (Fisher A78), 20 mg/L Na_2_MoO_4_ ⋅ 2 H_2_O (Acros Organics 446360250).

We note several technical issues that arose when constructing and testing the various CDMs. First, as mentioned previously, using high concentrations of some amino acids in the CDM often leads to formation of a precipitate (likely an amino acid metal salt complex) when stored for >48 hours. We found that this problem is exacerbated when preparing Wolfe’s minerals with metals sourced from different suppliers. Re-filtering the media to remove the precipitate did not inhibit growth of the lactobacilli tested (not shown). While this suggests that lower metal concentrations are tolerable for some strains, this may impede the growth of lactobacilli that exhibit more stringent mineral requirements.

Second, while decreasing amino acid concentration inhibits precipitation, this could present challenges when culturing lactobacilli that are deficient in amino acid synthesis. Indeed, some strains grown on nutrient rich agar plates such as De Man, Rogosa and Sharpe (MRS) agar do not grow well when inoculated directly into CDM_*L*_. We found that conditioning the strains by passaging them through a mixture of 50:50 MRS:CDM_*L*_ before 100% CDM_*L*_ greatly improved growth rate. For weak growers, we found that up to three passages in 100% CDM_*L*_ were required to facilitate robust growth.

### Optimization of the CDM for *Acetobacter* growth and co-culturing

**Fig 2:**
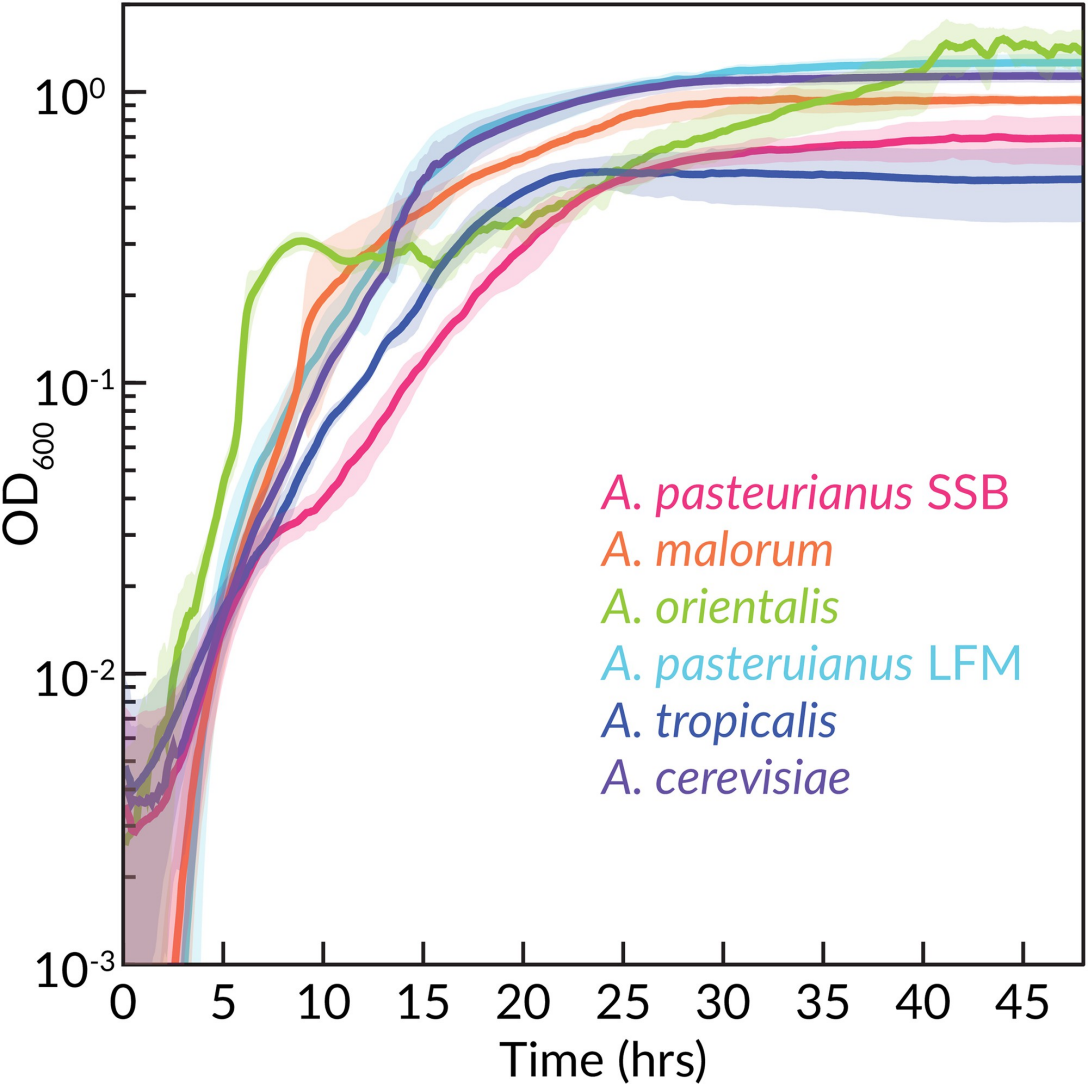
Growth of *Acetobacter sp.* isolates. *Acetobacter sp.* from several different sources (S2 Table in S1 File) were grown with CDM_*A*_ under aerobic conditions with continuous shaking. Solid lines represent means of 12 technical replicates. Shaded areas represent standard error on a 5-point rolling average. Time point OD_600_ readings were taken every 5 minutes.

To test the CDM’s ability to support growth of AAB, we incubated *A. pasteurianus* and *A. tropicalis* in CDM_*L*_, which produced extremely limited growth (S5 Table in S1 File). In an attempt to improve growth, we repeated the 10-fold additions experiment for *Lp* growth and found only ascorbate provided growth improvement (S4 Table in S1 File). Because ascorbate reacts with Wolfe’s minerals as evidenced by discoloration of the media after 48 h at room temperature, we use it optionally. Through a series of trials, we found that supplementing CDM_*L*_ with 50 mM D,L-lactic acid was sufficient to enable growth. This formulation, CDM_*A*_, supports an OD_600_ of ∼ 1.0 (∼ 10^9^ cells/mL) for the six *Acetobacter*s tested (Fig 2). We note that it was critical to supply adequate aeration to *Acetobacter*s by shaking and poking a 0.5 mm hole in the breathable sealing film over the plate. Increased shaking speed increased the clumping of the cultures, which appears as increased variance in the growth curves between replicates (Fig 2). Reducing the shaking speed reduced clumping but also reduced the growth rate. We also found that mannitol can serve as a carbon source for *Acetobacter*s.

**Fig 3:**
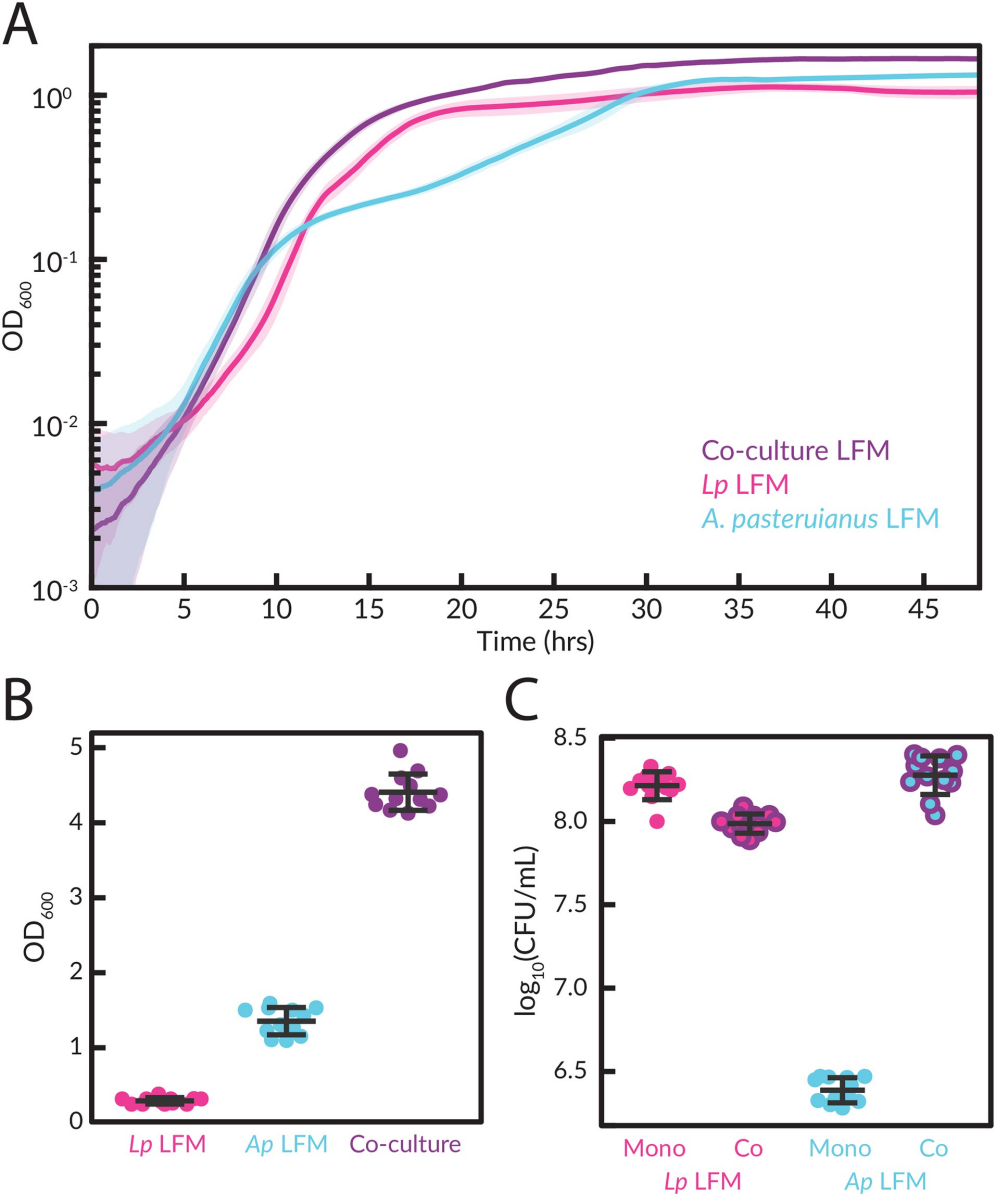
Co-culture growth of *Lp. plantarum* and *A. pasteurianus*. *Lp. plantarum* and *A. pasteurianus* were grown in co-culture in CDM_*A*_ with glucose and potassium acetate added. **(A)** Growth curves (solid lines) display means of 12 experimental replicates. Shaded areas represent standard error on a 5-point rolling average. Time point OD_600_ readings were taken every 5 minutes. **(B)** OD measurements were taken at the end of the growth curve after resuspension of the culture supernatant for each of the 12 replicates. Mean and standard deviation are shown. We note that the apparently 4-fold lower OD of *Lp. plantarum* in the resupsended culture versus the time course in **A** is likely due to settling of the cells and adherence to the plate during the time course. The CFU/mL counts are in **C**. **(C)** CFU/ml was counted by plating the resuspended cultures on MRS and MYPL for each of the 12 replicates. Mean and standard deviation shown.

For co-cultures with *Lp. plantarum* and *A. pasteurianus*, we add glucose and potassium acetate to CDM_*A*_ (Table 3). In these co-cultures, the OD_600_ approximated the combined OD_600_ for the two strains grown separately (Fig 3A) but reached an overall higher final yield of ≈ 4 versus ≈ 1 for *A. pasteurianus* and < 1 for *Lp. plantarum* (Fig 3B). By plating the co-culture on selective media (MRS for *Lp. plantarum* versus MYPL for *A. pasteurianus*), we found that *A. pasteurianus* increased growth yield from ∼ 10^6^ CFU/mL to ∼ 10^8^ CFU/mL while *Lp. plantarum* yield remained roughly constant at ∼ 10^8^ CFU/mL (Fig 3C), indicating that growth with *Lp. plantarum* provides an advantage to *A. pasteurianus* in these specific co-culture conditions.

**Table 3:** Composition of CDM_*A*_.

Stock solution #	Chemical	Final concentration (1x)
**Buffer and salts**		
1	MOPS	40 mM (1x)
1	K_2_HPO_4_	5 mM (1x)
1	NH_4_Cl	20 mM (1x)
1	Na_2_SO_4_	10 mM (1x)
**Metals**		
2	MgCl_2_ ⋅ 6 H_2_O	1 mM (1x)
2	MnCl_2_ ⋅ 4 H_2_O	0.05 mM (1x)
2	FeSO_4_ ⋅ 7 H_2_O	0.05 mM (1x)
**Carbon source**		
3	DL-Lactic acid	0.6 mM (1x)
3	Glucose or mannitol (optional)	125 mM (1x)
3	Ammonium acetate (optional)	10 mM
**Amino acids**		
4	Casamino acids	3 g/L
5	Cysteine-HCl	0.145 g/L
6	Tryptophan	0.05 g/L
7	**Wolfe’s vitamins**	1x
8	**Wolfe’s minerals**	1x

## Conclusions

The CDM formulated provides a tractable growth medium for the investigation of lactobacillus-*Acetobacter* community growth and metabolism under defined conditions so that genes and metabolites can be causally linked through experiments.

The CDM may also provide a starting point for investigation of more complex LAB and AAB communities, which are often observed in nature. We have investigated many *Lp. plantarum* strains and species of *Acetobacter* and found consistent growth. Due to the known species- and strain-dependence of lactobacilli growth, investigators of other LAB may find this CDM a useful starting point for devising new CDMs that support growth of other LAB species and genera. For instance, initial tests with *Ll. brevis* indicated weak but consistent growth when certain additives were supplied (S4 Table in S1 File).

The complex metabolic interactions in natural microbial communities have important impacts on their environments, including soils, oceans, and the guts of animals. Studying the fundamental biology of these interactions is facilitated by the use of CDMs and naturally low diversity microbial communities. For instance, with an optically clear CDM such as this one, experiments to combinatorically altering the chemical components are possible by running growth experiments in 96-well plates. We hope that this CDM for the *Drosophila* gut microbiome provides a foundation for the molecular characterization of the complex interactions in this naturally low diversity community.

## Supporting information

S1 File (PDF)
