# Supplementary material for: A chemically-defined growth medium to support Lactobacillus-Acetobacter sp. community analysis
Source: PLoS One. 2023 Oct 12;18(10):e0292585. doi: 10.1371/journal.pone.0292585 (PMC10569604; doi:10.1371/journal.pone.0292585)
Supplement: S1 File — (PDF) [file pone.0292585.s001.pdf]

Supplemental Materials:
A chemically-defined growth medium to support *Lactobacillus-Acetobacter sp.* community analysis.

Kevin Aumiller[1,2◑] Robert Scheffler[1◑] Eric T. Stevens[3◑] Zehra T. Güvener[4◑] Emily Tung[4] Anna B. Grimaldo[5] Hans K. Carlson[6] Adam M. Deutschbauer[5,6] Michiko E. Taga[5] Maria L. Marco[3] William B. Ludington[,1,2*]

1 Carnegie Institution of Washington, Department of Embryology, Baltimore, MD 21218, USA **2** Johns Hopkins University, Department of Biology, Baltimore, MD 21218, USA
3 University of California, Davis, Department of Food Science and Technology, Davis, CA 95616, USA
4 University of California, Berkeley, Molecular and Cell Biology Department, 94720, USA
5 University of California, Berkeley, Department of Plant and Microbial Biology, 94720, USA
6 Lawrence Berkeley National Laboratory, Department of Environmental Genomics and Systems Biology, Berkeley, CA 94720, USA

◑These authors contributed equally to this work.
* ludington@carnegiescience.edu

References

1. Savijoki K, Suokko A, Palva A, Varmanen P. New convenient defined media for [35S]methionine labelling and proteomic analyses of probiotic lactobacilli. Letters in Applied Microbiology. 2006;42(3):202–209. doi:10.1111/j.1472-765X.2005.01853.x.

Table 1. Supplemental List of chemicals used in this study

Compound	Vendor	Catalog number	Molecular weight
MOPS, Free acid, UltraPure	GoldBio	M790	209.26
K2HPO4	Fisher Scientific	P288	174.18
NaCl	Fisher Scientific	S271	58.44
NH4Cl	Fisher Scientific	A649	53.49
K2SO4	Sigma	P9458	174.26
MgCl2.6H2O	Fisher Scientific	BP214	203.31
MnCl2.4H2O	Sigma	M3634	197.9
FeSO4.7H2O	Sigma	F8633	278.01
L-Alanine	Sigma	A7627	89.09
L-Arginine	Sigma	A5131	210.66
Glycine	Sigma	G7126	75.07
L-Lysine	Sigma	L5626	146.19
L-Proline	Sigma	P0380	115.13
L-Histidine	Sigma	H8125	155.15
L-Serine	Sigma	S4500	105.09
L-Threonine	Sigma	T8625	119.12
L-Aspartic acid	Sigma	A9256	133.1
L-Asparagine	Sigma	A0884	132.12
L-Tyrosine	Sigma	T3754	181.19
L-Cysteine-HCl	Sigma	C1276	157.62
L-Valine	Sigma	V0500	117.15
L-Glutamic acid	Sigma	G1251	183.6
L-Tryptophane	Sigma	T0254	204.23
L-Phenylalanine	Sigma	P2126	165.19
L-Glutamine	Sigma	G3126	146.14
L-Leucine	Sigma	L8000	131.17
L-Isoleucine	Sigma	I2752	131.17
L-Methionine	Sigma	M9625	149.21
Ca-(D)-(+)-pantothenate	Sigma	C8731	238.27
Lipoic acid	Sigma	T1395	206.33
Nicotinic acid	Sigma	72309	123.11
Para-Aminobenzoic acid	Sigma	A9878	137.14
Pyridoxine-HCl	Sigma	P9755	205.64
Thiamine-HCl	Sigma	T4625	337.27
Biotin	Sigma	B4639	244.31
Ascorbic acid	Fisher Scientific	AA3623714	176.12
Folic acid	Sigma	F7876	441.4
Riboflavin	Sigma	R4500	376.36
Cyanocobalamin	Sigma	V6629	1355.37
Guanine	Sigma	G6779	151.13
Uracil	Sigma	U1128	112.09
Xanthine	Sigma	X4002	152.11
Adenine	Sigma	A2786	135.13
Glucose	Sigma	G8270	180.16
Fructose	Sigma	F3510	180.16
Acetate, sodium salt	Fisher Scientific	S210	90.08

Table 2. Supplemental Bacterial strains used in this study

Strain	Source	Original study
Lp. plantarum WF	wild *D. melanogaster isolate*	Obadia et al. 2017
Lp. plantarum NC8	fermented grass	Axelsson et al. 2012
Lp. plantarum NCIMB8826	human saliva	Hayward & Davis 1956
Lp. plantarum ATCC 8014	food	(Orla-Jensen) Bergey et al
Lp. plantarum LF	Canton-S *D. melanogaster*	Obadia et al. 2017
Lp. plantarum endoref	lab *D. melanogaster*	Storelli et al. 2011
Lp. plantarum SB001	lab *D. melanogaster*	Obadia et al. 2017
Lp. plantarum SSB	wild *D. melanogaster*	Hardy et al. 2018
Lp. plantarum WJL	lab *D. melanogaster*	Ryu et al 2008
Lp. plantarum K4	wheat sourdough starter	Yu et al 2021
Lp. plantarum 8.1	wheat boza	Yu et al 2021
Lp. plantarum W1.1	wheat flour teff injera	Yu et al 2021
Lp. plantarum B1.1	brown flour teff injera	Yu et al 2021
Lp. plantarum B1.3	brown flour teff injera	Yu et al 2021
Lp. plantarum T2.5	fermented tomatoes	Yu et al 2021
Lp. plantarum WS1.1	fermented tomatoes (spoiled)	Yu et al 2021
Lp. plantarum 1B1	cactus fruit (Opuntia ficus-indicia)	Tyler et al 2016
Lp. plantarum AJ11	fermented olives	Golomb et al 2013
Lp. plantarum BGM55	fermented olives inoculated with yeast	Golomb et al 2013
Lp. plantarum BGM37	olive fermentation brine	Golomb et al 2013
Lp. plantarum BGM40	fermented olives	Golomb et al 2013
Lp. plantarum EL11	fermented olives	Golomb et al 2013
Ll. brevis LF	isolated from lab D. melanogaster	Gould et al. 2018
Ll. brevis LF2	isolated from lab *D. melanogaster*	Obadia et al. 2017
Ll. brevis SSA	isolated from wild *D. melanogaster*	Hardy et al. 2018
Ll. brevis Di	isolated from wild *D. immigrans*	this study
A. orientalis	Canton-S isolate	Gould et al. 2018
A. pasteurianus LFM	Oregon-R isolate	Gould et al. 2018
A. pasteurianus SSB	isolated from lab *D. melanogaster*	this study
A. tropicalis	Oregon-R isolate	Gould et al. 2018
A. cerevisiae	wild *D. melanogaster*	this study
A. malorum	wild *D. melanogaster*	this study

Table 3. Supplemental Final growth yield values of strains grown in CDM with indicated fold-difference of the combined amino acids, vitamins, and nucleotides from the concentration of components listed in Table 1.

**Concentration**	***Lp. plantarum***	***Ll. brevis***	***A. pasteurianus***	***A. tropicalis***
2x	0.449 (sem 0.029)	0.060 (sem 0.005)	0.101 (sem 0.078)	0.072 (sem 0.005)
1x	0.236 (sem 0.016)	0.058 (sem 0.002)	0.151 (sem 0.014)	0.096 (sem 0.001)
0.5x	0.085 (sem 0.009)	0.031 (sem 0.001)	0.103 (sem 0.004)	0.093 (sem 0.004)

Table 4. Supplemental Growth yield values of strains grown in CDM from [1] with indicated additives at 10-fold excess

Additive	***Lp. plantarum***	***Ll. brevis***	***A. pasteurianus***	***A. tropicalis***
CDM no additives	0.238 (sem 0.002)	0.131 (sem 0.001)	0.103 (sem 0.020)	0.036 (sem 0.001)
Ala	0.218 (sem 0.009)	0.138 (sem 0.000)	0.199 (sem 0.000)	0.073 (sem 0.007)
Asn	0.229 (sem 0.019)	0.132 (sem 0.005)	0.136 (sem 0.112)	0.037 (sem 0.006)
Glu	0.317 (sem 0.011)	0.071 (sem 0.006)	0.030 (sem 0.004)	0.049 (sem 0.002)
Phe	0.285 (sem 0.033)	0.134 (sem 0.002)	0.062 (sem 0.040)	0.034 (sem 0.003)
Leu	0.269 (sem 0.109)	0.130 (sem 0.002)	0.025 (sem 0.003)	0.047 (sem 0.004)
Ile	0.228 (sem 0.001)	0.135 (sem 0.011)	0.064 (sem 0.023)	0.036 (sem 0.005)
Met	0.231 (sem 0.002)	0.123 (sem 0.000)	0.024 (sem 0.041)	0.040 (sem 0.001)
Val	0.251 (sem 0.044)	0.138 (sem 0.001)	0.032 (sem 0.003)	0.004 (sem 0.001)
Trp	0.877* (sem 0.535)	0.127 (sem 0.004)	0.128 (sem 0.059)	0.036 (sem 0.003)
Gln	0.287 (sem 0.007)	0.076 (sem 0.003)	0.000 (sem 0.002)	0.039 (sem 0.000)
Arg	0.118 (sem 0.030)	0.124 (sem 0.000)	0.085 (sem 0.021)	0.033 (sem 0.002)
Gly	0.217 (sem 0.005)	0.133 (sem 0.000)	0.156 (sem 0.040)	0.034 (sem 0.000)
Lys	0.213 (sem 0.000)	0.141 (sem 0.001)	0.099 (sem 0.013)	0.041 (sem 0.000)
Pro	0.298 (sem 0.016)	0.155 (sem 0.002)	0.058 (sem 0.020)	0.037 (sem 0.000)
His	0.268 (sem 0.019)	0.138 (sem 0.003)	0.118 (sem 0.083)	0.040 (sem 0.000)
Ser	0.296 (sem 0.008)	0.121 (sem 0.007)	0.054 (sem 0.002)	0.065 (sem 0.032)
Thr	0.257 (sem 0.025)	0.140 (sem 0.002)	0.046 (sem 0.011)	0.032 (sem 0.001)
Panthothenate	0.246 (sem 0.011)	0.138 (sem 0.002)	0.055 (sem 0.027)	0.027 (sem 0.009)
Lipoic acid	0.260 (sem 0.010)	0.111 (sem 0.004)	0.032 (sem 0.005)	0.033 (sem 0.000)
Cyanocobalamin	0.261 (sem 0.005)	0.136 (sem 0.000)	0.088 (sem 0.000)	0.045 (sem 0.003)
Nicotinic acid	0.218 (sem 0.007)	0.151 (sem 0.027)	0.074 (sem 0.002)	0.039 (sem 0.001)
para-Aminobenzoic acid	0.217 (sem 0.010)	0.155 (sem 0.028)	0.137 (sem 0.065)	0.038 (sem 0.004)
Pyridoxine	0.225 (sem 0.000)	0.135 (sem 0.001)	0.067 (sem 0.007)	0.038 (sem 0.004)
Thiamine	0.231 (sem 0.012)	0.136 (sem 0.001)	0.143 (sem 0.051)	0.037 (sem 0.000)
Biotin	0.299 (sem 0.009)	0.137 (sem 0.005)	0.075 (sem 0.002)	0.041 (sem 0.000)
Ascorbate	0.231 (sem 0.007)	0.088 (sem 0.052)	0.316# (sem 0.370)	0.050 (sem 0.000)
Folate	0.227 (sem 0.010)	0.139 (sem 0.001)	0.071 (sem 0.009)	0.041 (sem 0.000)
Riboflavin	0.230 (sem 0.012)	0.135 (sem 0.000)	0.187 (sem 0.013)	0.037 (sem 0.001)
Guanine	0.212 (sem 0.004)	0.136 (sem 0.000)	0.124 (sem 0.035)	0.042 (sem 0.005)
Uracil	0.267 (sem 0.000)	0.155 (sem 0.004)	0.066 (sem 0.019)	0.043 (sem 0.003)
Xanthine	0.243 (sem 0.007)	0.138 (sem 0.002)	0.069 (sem 0.018)	0.040 (sem 0.003)
Adenine	0.252 (sem 0.007)	0.133 (sem 0.001)	0.055 (sem 0.033)	0.044 (sem 0.002)
PO4	0.263 (sem 0.008)	0.208 (sem 0.000)	0.042 (sem 0.018)	0.037 (sem 0.002)
SO4	0.276 (sem 0.074)	0.132 (sem 0.000)	0.120 (sem 0.081)	0.042 (sem 0.001)
NH4	0.200 (sem 0.004)	0.159 (sem 0.002)	0.040 (sem 0.017)	0.036 (sem 0.003)
Mg	0.205 (sem 0.092)	0.154 (sem 0.039)	0.060 (sem 0.003)	0.039 (sem 0.002)
Mn	0.290 (sem 0.008)	0.157 (sem 0.003)	0.113 (sem 0.029)	0.057 (sem 0.020)
Fe	0.210 (sem 0.007)	0.157 (sem 0.001)	0.120 (sem 0.029)	0.042 (sem 0.001)
Na	0.177 (sem 0.032)	0.139 (sem 0.001)	0.164 (sem 0.085)	0.018 (sem 0.000)
Bicarbonate, 40 mM	0.347* (sem 0.019)	0.0135 (sem 0.001)	0.022 (sem 0.004)	0.038 (sem 0.000)

* confirmed increases *Lp* growth.

# confirmed increases *Ap* growth.

Table 5. Supplemental Growth yield values of strains grown in CDM from *Savijoki et al 2006* with indicated carbon sources.

**Strain**	**Gluc 0.65% Fruc 0.65% Acet 0.1%**	**Gluc 0.65% Fruc 0.65%**	**Gluc 1% Acet 0.1%**	**Fruc 1%**	**Gluc 1%**	**Acet 0.1%**
Lp. plantarum	–[1]	–	0.123	–	0.056	0.001
Ll. brevis	0.051	0.026	–	–	–	0.000
A. pasteurianus	–	–	–	0.026	–	0.014
A. tropicalis	–	–	0.021	–	0.044	0.021

1. − indicates condition not tested

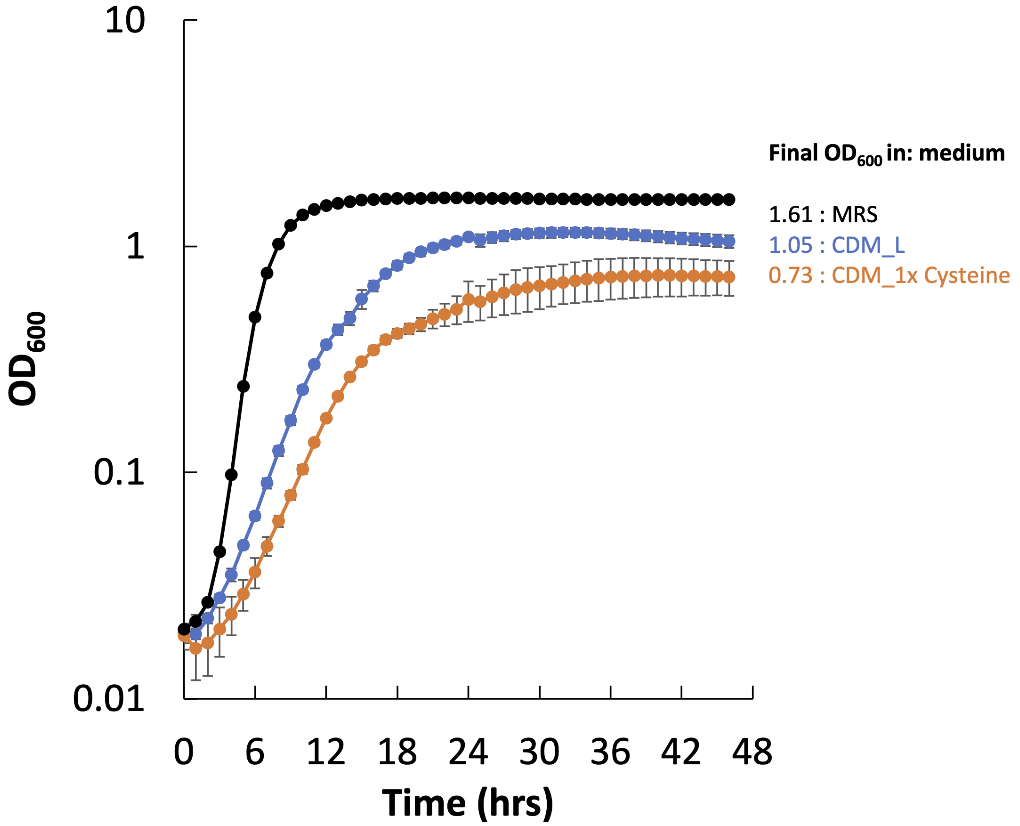

Fig 1. Supplemental Growth of *Lp. plantarum* NCIMB8826 in MRS and CDM$_L$ with different amounts of cysteine. Inoculation at 0.05 OD$_{600}$ from overnight cultures of MRS grown cells washed in PBS. Aerobic growth in 96 well plates with continuous shaking 12 technical replicates per growth curve. OD$_{600}$ readings each 1 hour. Error bars are standard error. Note that CDM$_L$ has 10x the cysteine of CDM$_L$ 1x cysteine.
